# Understanding the Limits of Patient Safety Classification Systems for Health Information Technology–Related Incidents

**DOI:** 10.2196/91783

**Published:** 2026-08-28

**Authors:** Md Shafiqur Rahman Jabin

**Affiliations:** 1 Department of Medicine and Optometry Linnaeus University Kalmar Sweden; 2 Faculty of Health and Social Care University of Bradford Bradford, West Yorkshire United Kingdom

**Keywords:** sociotechnical systems, knowledge representation, incident reporting, safety science, information infrastructure, organizational learning, sensemaking, complex adaptive systems, data standardization, epistemic limits

## Abstract

Patient safety classification systems are fundamental to surveillance, organizational learning, research, and governance because they enable adverse events and near misses to be organized into standardized categories for comparison and analysis. However, the increasing complexity of health information technology (HIT)–related patient safety incidents challenges the assumptions underpinning conventional classification approaches, as these incidents often emerge from dynamic, distributed, and evolving sociotechnical interactions rather than discrete, time-bounded events. In this Viewpoint, I argue that many of the challenges associated with classifying HIT-related patient safety incidents arise not simply from limitations of individual classification systems but from the inherent representational logic of classification itself. By viewing classification as a knowledge practice rather than merely a technical tool for organizing incident data, I contend that abstraction, boundary-setting, and standardization inevitably simplify complex sociotechnical processes and constrain how safety problems are represented, interpreted, and acted upon. I discuss 4 recurring representational limitations that characterize the application of patient safety classification systems to HIT-related incidents: fragmentation of sociotechnical interactions, loss of temporality and evolving processes, inadequate representation of scale and propagation across systems, and normalization of “use error” through simplified attribution of responsibility. These limitations can contribute to incomplete organizational learning, misaligned safety interventions, and challenges in interpreting and comparing classified patient safety data across health care settings. Rather than arguing against the continued use of patient safety classification systems, I propose that their strengths and limitations should be recognized simultaneously. Classification remains indispensable for surveillance, learning, and governance, but it should be interpreted as one component of a broader sociotechnical understanding of patient safety. Recognizing the representational limits of classification can support more reflexive interpretation of classification-based evidence and encourage complementary approaches that better capture the complexity of HIT-related patient safety.

## Introduction

### Classification as Infrastructure in Patient Safety

Classification systems are foundational to patient safety research, surveillance, and institutional learning. By transforming diverse and complex safety incidents into standardized categories, classification enables aggregation, comparison, and analysis across settings and over time. These systems underpin reporting infrastructures, research studies, quality improvement initiatives, and policy conversations, while shaping how patient safety problems are recognized and addressed [1-3].

### Diversity of Patient Safety Classification Approaches

Patient safety classification schemes have been developed in a variety of contexts and based on diverse design logics. The International Classification for Patient Safety (ICPS), a World Health Organization (WHO)–created framework, represents a global effort to harmonize safety terminology and incident constructs [3]. Beyond ICPS, there are classification efforts tailored to specific settings or incident types (eg, primary care safety classifications developed through the LINNAEUS [Learning in an International Network About Errors and Understanding Safety] collaboration [4] and medication error taxonomies such as the NCC MERP [National Coordinating Council for Medication Error Reporting and Prevention] taxonomy [3,5,6]). Moreover, structured frameworks, such as human factors taxonomies, provide alternative logics for organizing contributing factors associated with safety events [7-9]. These varied systems share a core commitment: to render unsafe care legible through predefined categories and relationships.

### Why Health Information Technology Challenges Classification

The expansion of health information technology (HIT) into clinical work has strained conventional classification logic. HIT-related safety incidents often involve sociotechnical interactions, distributed causality, temporal processes, and emergent behaviors that are not easily reducible to discrete event types and fixed causal categories [10,11]. Although HIT-specific classification efforts (eg, adaptations of Health Information Technology Classification System (HIT-CS) and classifications that integrate technology and device issues) have attempted to capture these phenomena, persistent difficulties in representing HIT-related incidents suggest deeper epistemic limits in how classification systems encode knowledge [1,12].

Although HIT-specific and HIT-adapted classification schemes have been developed to address these challenges, recurring representational difficulties suggest that the problem may not lie solely in the design or implementation of particular frameworks [13]. For example, duplicate medication orders associated with computerized physician order entry (CPOE) systems have been reported as medication-related or HIT-related patient safety incidents, yet such classifications do not fully represent the interacting software, clinical workflow, human, and organizational factors that contributed to their occurrence [14,15].

### From Classification Design to Classification Limits

Prior work on HIT-related patient safety has addressed methodological challenges in studying technology-associated incidents and articulated interpretive perspectives on HIT-related risk. Building on this foundation, the present paper shifts attention from how incidents should be classified to what classification systems, by their nature, can and cannot represent. By making these inherent limits explicit, this Viewpoint clarifies the constraints on learning from classified safety data and suggests implications for future research in HIT safety.

### Perspective Presented in This Viewpoint

This Viewpoint examines the inherent limits of patient safety classification systems when applied to HIT-related incidents by considering classification as a representational practice rather than simply a technical tool. I argue that 4 recurring representational limitations help explain why patient safety classification systems struggle to adequately represent HIT-related incidents: (1) fragmentation of sociotechnical interactions, (2) loss of temporality and evolving processes, (3) inadequate representation of scale and propagation across systems, and (4) normalization of “use error” through simplified attribution of responsibility. Rather than proposing a new taxonomy or evaluating existing classification systems, this paper examines classification itself as an epistemic practice [16-19]. Importantly, sociotechnical perspectives on HIT safety are well established in the literature, and the present Viewpoint builds on this body of work by examining how classification systems interact with these broader sociotechnical perspectives.

This Viewpoint is intended for patient safety researchers, HIT researchers, patient safety classification developers, health care quality and safety professionals, and policymakers involved in the design, interpretation, and use of patient safety classification systems.

## Classification as a Representational Lens: Why Taxonomies Shape Interpretation Rather Than Merely Describe Events

### Classification as Knowledge Production

Patient safety classification systems are often presented as neutral tools for organizing information about adverse events and near misses. In practice, however, classification operates as a representational lens that actively shapes how safety problems are understood [1,6,20]. By defining categories and boundaries, classification systems shape what is recognized as an incident, how causality is attributed, and which dimensions of harm are foregrounded or obscured [6,20,21].

The term “system” is used in several ways throughout this paper. Specifically, it may refer to the clinical work system, the HIT system, the incident-reporting system, or the patient safety classification system. Unless otherwise specified, this perspective focuses on classification systems as representational frameworks while recognizing their interaction with broader sociotechnical and reporting systems.

### Abstraction and Standardization

At a fundamental level, all classification systems rely on abstraction and standardization. Classification makes complex phenomena easier to analyze by reducing them to predefined units. These units may include event types, contributing factors, or outcomes that can be applied consistently across cases. This abstraction enables aggregation and comparison across cases, supporting patient safety surveillance and learning. At the same time, abstraction necessarily compresses detail, strips away contextual nuance, and stabilizes fluid processes into discrete representational forms [21-23].

### Boundary-Setting and Assumptions About Causality

Classification systems also impose boundaries. They delineate what counts as an “incident,” what falls within the scope of patient safety, and where responsibility or causation is presumed to reside. These boundaries are embedded in category definitions, hierarchies, and exclusion criteria [21-24].

### Classification, Sensemaking, and Governance

Classification influences not only data organization but also sensemaking and governance. Classified data travel across organizational and institutional contexts, informing research syntheses, quality improvement initiatives, and policy decisions [25,26]. As categories are reused and institutionalized, they can stabilize particular narratives about safety problems while marginalizing others. Over time, this can create feedback loops in which certain types of incidents are repeatedly recognized and addressed, while others remain poorly articulated or invisible [25,27,28].

### Inherent Limits of Classificatory Work

These characteristics of classification, including abstraction, boundary-setting, and stabilization, are not flaws but constitutive features of classificatory work. As such, they introduce limits that cannot be fully eliminated through refinement or expansion of taxonomies [23,25]. Adding categories, increasing granularity, or tailoring classifications to specific domains may improve representation of particular incident features. However, these refinements do not overcome the underlying logic of classification, which continues to stabilize complex sociotechnical processes into discrete, comparable categories [28]. Instead, they redistribute rather than eliminate representational constraints [27].

**Figure 1:**
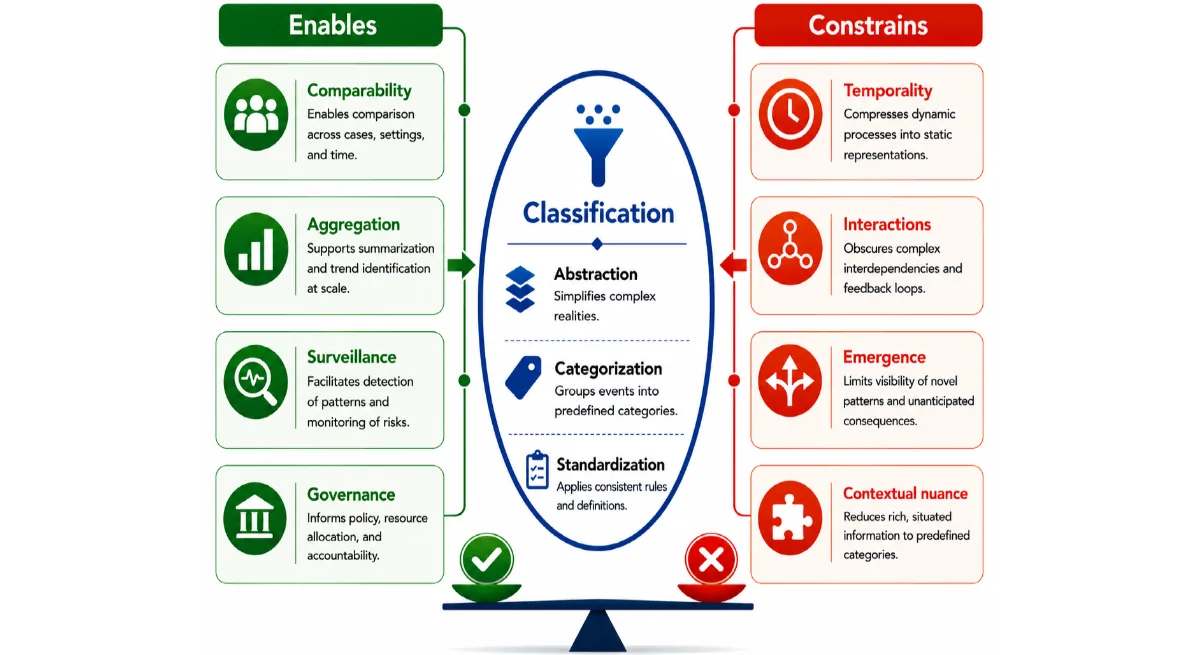
Representational trade-offs in patient safety classification.

In this paper, the term epistemic limits refers to constraints that originate from the fundamental logic of classification itself. These limits differ from design trade-offs, methodological limitations, or pragmatic constraints associated with reporting systems. Design trade-offs involve decisions about how a classification system is constructed or executed, while methodological limitations stem from analytic techniques or data availability. Pragmatic constraints reflect the institutional or operational needs of incident-reporting systems. In contrast, epistemic limits arise from the core features of classification as a knowledge practice, especially abstraction, boundary-setting, and stabilization, which inevitably convert complex, evolving sociotechnical processes into discrete, comparable categories. These limits, therefore, endure across various taxonomies and implementation settings. Figure 1 illustrates the representational trade-offs inherent in patient safety classification. The same structural properties that enable standardized reporting and organizational learning also constrain the representation of complex sociotechnical processes.

Figure 1 illustrates how abstraction, categorization, and standardization simultaneously enable organizational learning while constraining the representation of complex sociotechnical processes.

### Implications for a Sociotechnical Perspective on Patient Safety

**Figure 2:**
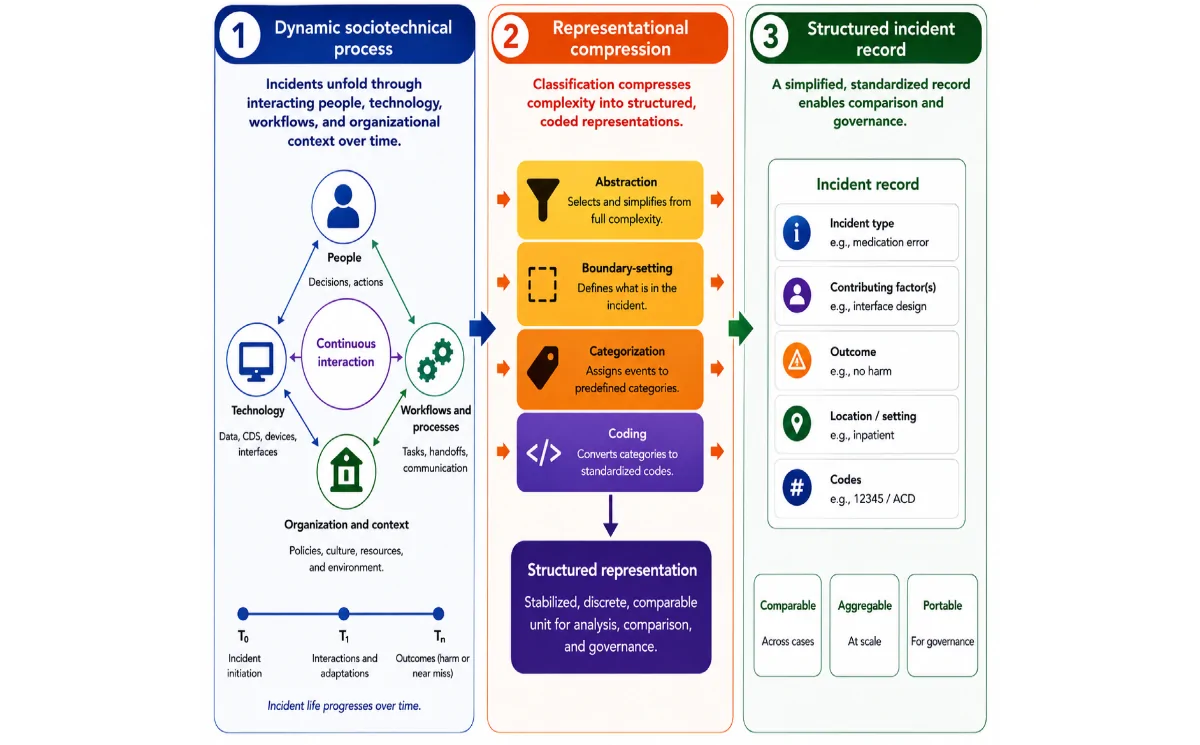
Representational compression in patient safety classification. CDS: clinical decision support; EHR: electronic health record.

Understanding classification as a representational lens is essential for examining patient safety in complex sociotechnical settings. In such contexts, the tension between the need for standardized representation and the reality of distributed, evolving processes becomes particularly pronounced [18,29,30]. Figure 2 illustrates representational compression in patient safety classification, whereby complex sociotechnical processes are transformed into discrete, standardized incident representations that support recording, categorization, comparison, and analysis.

Taken together, Figure 2 illustrates the core argument of this paper: the difficulties encountered in applying patient safety classification systems to HIT-related incidents arise from fundamental properties of classification itself. By converting evolving sociotechnical processes into discrete, stabilized representations, classification simultaneously enables learning at scale and imposes limits on what can be known, compared, and acted upon.

## Overview of Patient Safety Classification Approaches Relevant to HIT

### Illustrative Classification Traditions and Design Logics

Patient safety classification systems have been developed across diverse institutional, clinical, and analytic contexts. Although they differ in scope, purpose, and intended users, they share common representational commitments, relying on categorization, abstraction, and standardization to render safety incidents legible for analysis, comparison, and organizational learning [20,30,31]. The following examples illustrate how these shared design logics shape the representation of HIT-related safety problems.

### Global and General Patient Safety Frameworks

Global patient safety classification efforts aim to establish common conceptual foundations across health care systems. The ICPS was developed to harmonize terminology and incident constructs internationally [23,32,33].

Such frameworks are deliberately generic, prioritizing breadth and interoperability over contextual specificity. Consequently, they typically conceptualize safety incidents as discrete events that can be decomposed into causal elements and outcomes. Although this design supports aggregation and high-level analysis, it also embeds assumptions about temporality, agency, and system boundaries that become consequential when applied to complex sociotechnical phenomena [1,23,32,33].

### Reporting-Oriented Classification Systems

Many patient safety classifications are embedded in incident-reporting infrastructures and shaped by practical requirements for data collection, aggregation, and governance. In the United States, the Agency for Healthcare Research and Quality (AHRQ) Common Formats provide standardized categories for reporting adverse events, near misses, and unsafe conditions across care settings [25,34].

Reporting-oriented systems often emphasize clarity, usability, and consistency, favoring predefined categories that frontline reporters can apply. HIT-related issues are typically incorporated as contributing factors or device-related elements rather than as evolving sociotechnical processes [1,25,34].

### Domain-Specific and Error-Focused Taxonomies

Some classification systems focus on specific clinical domains or types of harm, such as medication safety. The taxonomy developed by the NCC MERP categorizes medication errors by the stages of the medication-use process and the severity of patient harm [1,5].

Although not specifically designed for HIT, such systems are frequently used for incidents involving electronic prescribing, decision support, and medication administration technologies. In doing so, they often normalize distinctions between “use error” and system malfunction, reflecting broader classificatory tendencies to localize error within identifiable actions rather than within distributed interactions [3,5].

### HIT-Specific and HIT-Adapted Classification Schemes

In response to growing recognition of technology-related risk, HIT-specific classification schemes have been developed to capture incidents involving electronic health records, clinical decision support, and other digital tools. These systems typically expand contributing-factor categories to include software design, interface issues, workflow mismatches, and organizational context. They are often explicitly informed by sociotechnical perspectives [10,35-37].

Despite this specialization, HIT-focused classifications retain core classificatory features shared with broader patient safety taxonomies. Incidents are still represented as bounded events, categorized according to predefined dimensions, and analyzed through stabilized causal structures. Consequently, these systems illuminate how far domain-specific adaptation can go without escaping the underlying logic of classification [10,18,38].

Recently, classification systems have incorporated more detailed categories, mechanisms for linking related events, and structured narrative components to better capture temporal dynamics and sociotechnical interactions [39,40]. Although these developments improve representation, they do not overcome the representational limitations inherent in classification as a mode of organizing patient safety information.

### Local and Adapted Classification Approaches

Beyond formal frameworks, many health care organizations and research teams develop local or adapted classification schemes to address perceived gaps in existing systems. These may involve extending categories, reinterpreting definitions, or combining elements from multiple frameworks [6,13,20,41].

The proliferation of adapted schemes underscores both the flexibility and the limits of classification. Efforts to tailor systems to local sociotechnical realities frequently reintroduce familiar representational challenges, suggesting that these challenges are not confined to particular taxonomies but arise from shared classificatory commitments [6,22,35,36].

Table 1 summarizes the illustrative classification design logics discussed in this section and highlights the shared representational commitments that shape the rendering of HIT-related safety problems.

**Table 1:** Illustrative classification design logics and their representational commitments.

Classification design logic	Primary unit of analysis	Assumptions about causality	Treatment of temporality	Typical handling of HIT^a^
Global patient safety frameworks	Discrete incident	Linear or decomposable	Event-based	Contributing factor
Reporting-oriented systems	Reportable event	Action- or device-linked	Snapshot at report time	Embedded category
Domain-specific taxonomies	Process step or error	Localized failure	Stage-based	Use error or malfunction
HIT-specific schemes	Incident with technical context	Sociotechnical (bounded)	Limited process capture	Expanded factors

^a^HIT: health information technology.

## Why Classification Struggles to Represent HIT-Related Incidents

### Overview

Patient safety classification systems vary in scope, purpose, and design. However, when applied to HIT-related incidents, they often encounter similar representational difficulties. These difficulties do not arise primarily from isolated design choices or implementation failures. Instead, they reflect recurrent representational tensions that emerge from the structural logic of classification itself [1,18].

**Figure 3:**
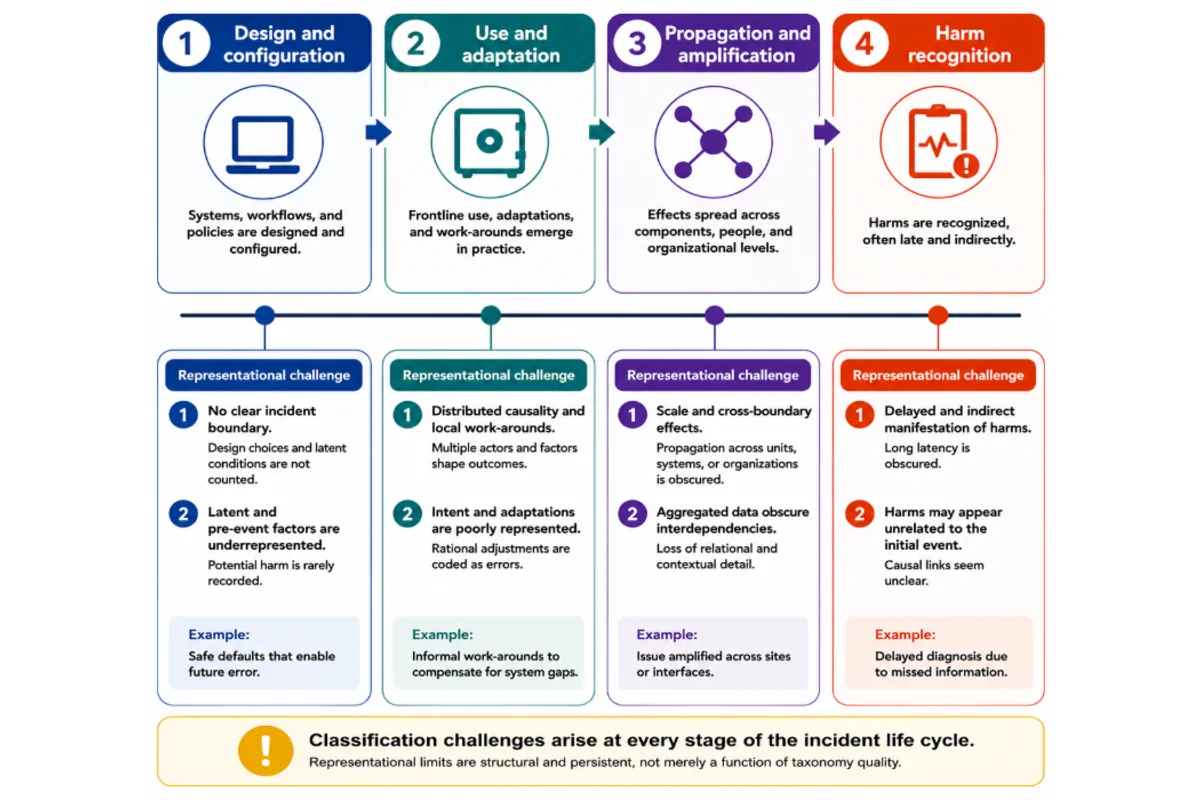
Representational challenges across the life cycle of health information technology–related incidents.

To illustrate the representational challenges discussed in this section, consider a simplified example involving a duplicate medication order generated through CPOE [14]. A physician enters a medication order, but due to interface design and workflow constraints, the system allows the same order to be entered twice. The duplicate order is later detected and corrected before it can cause harm. Depending on the classification system used, this incident might be coded as a medication error, a user-entry mistake, a workflow issue, or a software design problem [14,42,43]. Figure 3 illustrates where patient safety classification systems encounter recurring representational challenges across the life cycle of HIT-related incidents, from system design and clinical use to propagation and recognition of harm.

### Fragmentation of Sociotechnical Interactions

HIT-related safety incidents typically emerge from interactions among multiple system components, including software functionality, clinical workflows, organizational routines, and professional judgment. Classification systems, however, require incidents to be decomposed into discrete elements, such as event types, contributing factors, or actor roles, to render them categorizable [18,44]. In the CPOE duplicate-order example described previously, the incident could be attributed to user error, interface design, or workflow conditions, depending on the classificatory structure, illustrating how sociotechnical interactions become fragmented across multiple categories [14,42,43].

This decomposition breaks sociotechnical interactions into separate components. Consequently, the relational dynamics through which risk emerges may become less visible. Interdependencies between technology and practice are often redistributed across multiple categories, weakening the visibility of interaction effects. Consequently, the classified representation may suggest a set of parallel contributing factors rather than a coupled sociotechnical process [45,46].

This fragmentation is not an error of representation but a consequence of classificatory abstraction. Classification systems are designed to isolate elements that can be consistently named and compared, even when the phenomena of interest are fundamentally relational. For HIT-related incidents, this leads to representations that understate the role of interaction and overstate the independence of contributing components [18,26,46].

### Loss of Temporality and Process

Many HIT-related safety problems unfold over extended periods. Design decisions made during system configuration may interact with evolving patterns of use, work-arounds, and adaptation before contributing to harm that becomes apparent only much later. Classification systems, however, typically represent incidents as temporally bounded events, anchored to a moment of recognition or reporting [15,46,47]. In the duplicate-order example, the classification record may capture the moment the error was detected, while the preceding sequence of interface interactions and workflow adaptations that enabled the duplication remains largely invisible [14,42,43].

This event-based representation collapses trajectories into snapshots. Temporal sequencing, latency, and cumulative effects are difficult to encode within categorical structures that privilege discrete occurrences. Even when temporal fields are included, they rarely capture how risks develop, propagate, and transform over time [47].

The loss of temporality limits classification systems’ ability to represent HIT-related incidents as processes rather than outcomes. Consequently, downstream analysis may focus on proximal events while obscuring the longer-term dynamics through which sociotechnical risk accumulates [15,47].

Several patient safety classification systems have attempted to address these issues by introducing mechanisms intended to capture temporal and relational dynamics [48,49]. Although these features improve representation, they do not eliminate the underlying constraint that classification ultimately requires incidents to be stabilized into discrete, comparable categories.

### Poor Handling of Scale and Propagation

HIT-related incidents frequently propagate beyond the local context in which they originate. A configuration error, interface design issue, or workflow mismatch may affect multiple users, units, or care settings, with effects that scale unevenly and interact with local practices [46,47]. If similar configurations or interface conditions exist across multiple clinical units, duplicate orders may recur, yet classification systems may record them as independent incidents rather than manifestations of a shared systemic issue [12,50].

Classification systems, however, are typically organized around localized incident units. They privilege representations that tie incidents to specific locations, actors, or moments, making it difficult to capture cross-boundary propagation or system-wide effects. When scale is represented, it is often treated as an attribute of individual incidents rather than as a dynamic property of sociotechnical systems [1,51].

This mismatch between localized incident units and distributed effects constrains the conceptual reach of classification-based data. Propagation is rendered as repetition rather than as a feature of system structure, limiting insight into how HIT-related risks amplify across contexts [46,47].

### Normalization of “Use Error”

A recurrent feature of patient safety classification systems is the distinction between technical failure and human or “use” error. In HIT contexts, this distinction often becomes a default mode of representation, particularly when classification categories prioritize observable user actions over design-use coupling [11,52,53].

By treating “use error” as a categorical outcome, classification systems may normalize interpretations that assign responsibility to individual behavior, even when user actions are shaped by interface design, workflow constraints, or organizational pressures. Over time, this can reinforce a conceptual framework that treats sociotechnical misalignment as user deviation rather than as a property of system design [10,11,52].

This normalization effect does not require explicit attribution of blame. It emerges from the repeated use of categories that foreground action over interaction and outcome over process. As such, it reflects a deeper classificatory tendency to individualize causality in contexts where agency is distributed [1,38,51].

### Representational Limitations Are Structural, Not System-Specific

Taken together, these breakdowns illustrate that the difficulties classification systems encounter with HIT-related incidents are not confined to particular taxonomies or domains. Fragmentation, loss of temporality, scale mismatch, and normalization of use error recur across classification approaches precisely because they arise from shared classificatory commitments [54,55].

Efforts to address these issues through increased granularity or domain-specific adaptation may shift how representational limitations appear, but they do not eliminate the underlying tension between standardized representation and sociotechnical complexity [1,38,51].

The increasing use of large language models (LLMs) and natural language processing (NLP) for analyzing unstructured patient safety reports does not fundamentally eliminate the epistemic limits discussed in this Viewpoint. Although these technologies may improve the extraction, organization, and classification of information from free-text narratives, they ultimately produce structured representations that remain subject to abstraction, boundary-setting, and representational compression [40,54,56]. Consequently, automated text classification may enhance the efficiency and consistency of incident analysis, but it does not overcome the inherent limits of classification as a knowledge practice. Instead, it may automate and scale the same structural constraints that shape how complex sociotechnical incidents are represented and interpreted.

Table 2 summarizes the 4 recurring representational limitations discussed in this Viewpoint and their implications for the classification of HIT-related incidents.

**Table 2:** Recurring representational limitations in classifying health information technology–related incidents.

Representational limitations	What is lost or distorted?	Why does classification produce this limit?
Sociotechnical fragmentation	Interactions and dependencies	Forced attribution to components
Loss of temporality	Incident trajectories	Event-based representation
Scale and propagation	System-wide effects	Localized incident units
Normalization of use error	Design-use coupling	Person-centered categories

## Consequences for Research and Practice

### Overview

The representational constraints described in the previous section have important consequences for how patient safety data are interpreted, compared, and acted upon in both research and practice [1,57].

Classification-based incident data rarely function as the sole basis for safety improvement. Instead, they typically serve as starting points for root cause analysis, sociotechnical assessment, and organizational learning. In HIT, these investigations are often informed by sociotechnical frameworks such as the SAFER (Safety Assurance Factors for Electronic Health Record Resilience) Guides and related evaluation methods for electronic health record safety [18,56,58].

### Misleading Learning and Partial Explanations

Classification systems support learning by enabling aggregation and comparison across incidents. However, when HIT-related safety problems are represented as fragmented, temporally bounded events, the resulting learning may be partial or misleading. Aggregated patterns can obscure the sociotechnical interactions and trajectories through which harm emerges, encouraging interpretations that privilege proximal causes over longer-term or distributed dynamics [1,25,57]. When such interactions are flattened into discrete categories, learning may focus on symptoms rather than on the structural conditions that shape risk [1,25,57].

### Misaligned Interventions and Corrective Actions

The way incidents are classified influences not only interpretation but also intervention. When classified representations foreground individual actions or isolated failures, they can channel responses toward training, compliance, or local fixes, even when underlying issues are rooted in system design or organizational structure [30,47].

In HIT contexts, interventions derived from classification-based analyses may therefore focus on user behavior while leaving design-use mismatches or workflow constraints unaddressed. Over time, this can lead to repeated corrective efforts that fail to engage with the sources of sociotechnical risk, reinforcing cycles of reporting without commensurate improvement [30,46].

### Comparability Problems Across Studies and Settings

One of the primary motivations for classification is to enable comparability across studies, organizations, and jurisdictions. However, when HIT-related incidents are forced into stabilized categories that inadequately capture sociotechnical complexity, apparent comparability may mask substantive differences in how incidents unfold across contexts [22,33].

Differences in local workflows, system configurations, and organizational practices can lead to divergent interpretations of similar classified categories. Consequently, studies drawing on classified incident data may appear comparable at the level of codes while differing substantially in what those codes represent in practice. This complicates synthesis across studies and limits the interpretability of comparative findings in HIT-related patient safety research [22,23,45].

### Consequences as Structural, Not Correctable by Refinement

Taken together, these consequences indicate that the challenges associated with classifying HIT-related incidents are structural rather than contingent. Although greater granularity, additional categories, and domain-specific adaptations may improve representation, they do not eliminate the fundamental trade-offs inherent in classification-based representation [18,22].

Recognizing these consequences is not an argument against classification but a call for interpretive caution. Classification systems remain indispensable for surveillance, aggregation, and governance, yet their outputs should be understood as partial representations shaped by underlying design logics. Without this perspective, classification-based knowledge may be overextended beyond its representational limits [18,22].

## Implications for Future Research and Interpretation

### Overview

Recognizing the structural limits of classification systems has important implications for how future analyses of HIT-related patient safety are conceptualized and interpreted. These implications are particularly relevant for researchers analyzing patient safety datasets, HIT safety specialists interpreting incident reports, and health care organizations using classification-based data to guide quality improvement or governance activities [1]. Rather than seeking a single, more comprehensive classification framework, future research may benefit from approaches that explicitly acknowledge the partial and situated nature of classificatory representations [25,59]. Classification systems are best viewed as one part of a larger safety analysis ecosystem rather than as complete representations of complex sociotechnical events.

### Why No Single Framework Is Sufficient

The recurrent representational constraints discussed in this Viewpoint suggest that no single classification framework can fully capture the complexity of HIT-related safety incidents. Sociotechnical phenomena are characterized by interaction, emergence, and temporal evolution, whereas classification systems necessarily stabilize phenomena into bounded, comparable units. This tension cannot be resolved through additional categories or greater granularity alone [1,51,60]. Consequently, classification-based data should be interpreted as partial representations of complex sociotechnical processes. Although such data remain essential for identifying patterns of harm, prioritizing interventions, and comparing safety performance across settings, they should be interpreted alongside complementary approaches that preserve temporal, organizational, and sociotechnical context.

Efforts to design more inclusive or detailed taxonomies may improve the visibility of certain dimensions of risk, but they simultaneously introduce new boundary problems and interpretive constraints. Consequently, attempts to “solve” classificatory limitations by expanding the framework risk reproducing the same representational trade-offs in different forms. These observations suggest the value of a more reflexive interpretation of classification-based safety data, particularly in sociotechnical contexts [1,51,60]. Recognizing the limits of classification can help analysts interpret incident data more cautiously, particularly when drawing conclusions about causality, system-level risk, or the effectiveness of interventions.

### The Need for Reflexive Use of Classification Data

Future patient safety analyses that rely on classified incident data should adopt a reflexive stance toward the categories they use. Rather than treating classifications as transparent representations of underlying events, analysts should consider how category definitions, incident boundaries, and causal structures shape what becomes visible and actionable [18,51]. Without this reflexive orientation, there is a risk that classification outputs will be overinterpreted as direct reflections of sociotechnical reality [26,45,60].

### Layered and Complementary Analytic Perspectives

Given the limits of any single representational approach, future research on HIT-related patient safety may benefit from layered perspectives that combine classification-based data with complementary approaches. These may include qualitative inquiry, sociotechnical modeling, process tracing, or narrative analysis, each of which foregrounds dimensions of interaction, temporality, context, and emergence that classification tends to suppress [1,51,61].

Classification systems may remain central for surveillance and aggregation, while complementary approaches are used to explore interaction, temporality, and emergence. Together, these perspectives can support a more nuanced understanding of sociotechnical risk without relying on a single conceptual framework [1,51,61].

### From Representational Limits to Analytic Awareness

The implications of this Viewpoint are not methodological prescriptions but a call for more reflexive interpretation of classification-based evidence. By making the representational limits of classification explicit, future research can better align its interpretations and inferences with what classification-based data can reasonably support. This awareness can help prevent overgeneralization, misplaced attribution, and premature conclusions about causality or the effectiveness of interventions [1,51,62].

Treating classification as one analytic lens among many, rather than as a definitive account of safety, may enable more responsible and context-sensitive interpretations of HIT-related patient safety data [1,51,62].

## Discussion

Patient safety classification systems have become indispensable for surveillance, organizational learning, research, and governance because they provide a standardized means of organizing complex safety information. However, I argue that their value should not obscure an important limitation: classification is not simply a neutral mechanism for recording events but a representational practice that shapes how safety problems are perceived, interpreted, and addressed [13,51]. This distinction is particularly important for HIT-related patient safety incidents, which emerge through dynamic interactions among technologies, clinical workflows, organizational contexts, and human decision-making rather than as discrete, isolated events [35,36].

From this perspective, the recurring representational limitations discussed in this Viewpoint should not be understood as shortcomings of particular classification systems or reporting infrastructures. Rather, they reflect the inherent logic of classification itself. Processes such as abstraction, boundary-setting, and standardization make comparison and aggregation possible, but they also simplify the complexity of sociotechnical systems. Consequently, aspects of HIT-related incidents, including evolving interactions, temporal dynamics, distributed causation, and contextual influences, may be compressed or excluded during classification. These trade-offs are intrinsic to representational systems and cannot be eliminated solely by developing more detailed or domain-specific taxonomies.

Recognizing these representational limits has important implications for patient safety research and practice. Classification-based datasets are often interpreted as comprehensive accounts of safety events and are subsequently used to inform organizational learning, quality improvement, policy development, and regulatory decision-making. However, when classified data are treated as complete representations of underlying events, they may support only a partial understanding of complex safety problems. This can contribute to interventions that address visible categories of harm while overlooking the sociotechnical conditions that produced them, and it may complicate comparisons across organizations and health care settings where contextual factors differ substantially.

My argument is therefore not that patient safety classification systems should be replaced or that more comprehensive classification schemes will fully resolve these challenges. Greater granularity and HIT-specific refinement can undoubtedly improve representation, but they cannot eliminate the fundamental trade-offs inherent in any attempt to translate complex, evolving practices into stable categories. Instead, classification should be understood as one conceptual perspective among several, with its outputs interpreted alongside complementary approaches that preserve temporal, organizational, and sociotechnical context.

Although this Viewpoint focuses on HIT-related patient safety, the underlying argument extends more broadly to contemporary health care. As digital technologies become increasingly embedded within clinical practice and health systems continue to rely on standardized data infrastructures for surveillance, benchmarking, accountability, and learning, similar representational tensions are likely to emerge wherever complex practices are reduced to standardized forms of knowledge. Appreciating these tensions is therefore essential not only for interpreting patient safety data but also for understanding the broader strengths and limitations of data-driven governance in health care.

The contribution of this Viewpoint is not to advocate for another patient safety classification framework. Rather, it is to clarify the epistemic boundaries within which classification-based patient safety data should be interpreted. By making the limits of classification explicit, I encourage a more reflexive use of classification systems that acknowledges both their indispensable role in surveillance, organizational learning, and governance and their inability to fully capture the complexity of HIT-related patient safety. Such an approach supports a more balanced interpretation of classification-based evidence and reinforces the importance of combining standardized classification with complementary sociotechnical perspectives when investigating, learning from, and responding to digital health safety incidents.

### Conclusions

This Viewpoint argues that patient safety classification systems are indispensable for organizing, interpreting, and learning from adverse events and near misses. However, when applied to HIT-related incidents, they encounter recurring representational limitations that cannot be fully overcome through refinement or domain-specific adaptation. These limitations arise from the inherent properties of classification itself (ie, abstraction, boundary-setting, and stabilization), which transform dynamic sociotechnical processes into stable, comparable categories.

Viewing classification as a representational lens, rather than as a neutral container of safety information, helps explain why fragmentation of sociotechnical interactions, loss of temporality, inadequate representation of scale and propagation, and normalization of use error recur across different classificatory traditions. These limitations are not shortcomings of individual classification systems but predictable consequences of representing complex, evolving sociotechnical phenomena through standardized categories.

The contribution of this Viewpoint is not to advocate for another classification framework but to clarify the epistemic boundaries within which classification-based patient safety data should be interpreted. Recognizing both the strengths and the representational limits of classification supports a more reflexive use of classification-based evidence while reinforcing the indispensable role of classification systems in surveillance, organizational learning, research, and governance.

## Data Availability

No new data were generated or analyzed in this study. This Viewpoint paper is informed by previously published studies and the author’s prior research. As such, data sharing is not applicable.

## Abbreviations

AHRQ: Agency for Healthcare Research and Quality
CPOE: computerized physician order entry
HIT: health information technology
HIT-CS: Health Information Technology Classification System
ICPS: International Classification for Patient Safety
LINNAEUS: Learning in an International Network About Errors and Understanding Safety
LLM: large language model
NCC MERP: National Coordinating Council for Medication Error Reporting and Prevention
NLP: natural language processing
SAFER: Safety Assurance Factors for Electronic Health Record Resilience
WHO: World Health Organization
